# Upregulation of MiR-196a promotes cell proliferation by downregulating p27^kip1^ in laryngeal cancer

**DOI:** 10.1186/s40659-016-0100-9

**Published:** 2016-09-27

**Authors:** Cheng Jin, Yi Zhang, Jiping Li

**Affiliations:** Department of Otorhinolaryngology, School of Medicine, Ren Ji Hospital, Shanghai Jiao Tong University, No.145 Pujian Road, Shanghai, 200127 China

**Keywords:** miR-196a, p27^kip1^, Laryngeal cancer

## Abstract

**Background:**

Accumulating evidence has confirmed that miR-196a plays a critical role in tumorigenesis and tumor progression in a variety of cancers. It has been demonstrated that miR-196a is highly up-regulated in laryngeal cancer by miRNA profiling analysis. However, the functional mechanism of miR-196a in laryngeal cancer remains unclear. This study aims to explore the mechanism of miR-196a in laryngeal cancer.

**Methods:**

In the present study, we conducted qPCR analysis of miR-196a expression in human laryngeal cancer and showed that miR-196a was overexpressed in tumor-derived samples and laryngeal cancer cell lines compared with matched normal controls. Further functional analysis of miR-196a demonstrated that the inhibition of miR-196a could inhibit laryngeal cell-cycle progression and proliferation in vitro. Luciferase reporter assay and western blot confirmed that miR-196a directly targeted p27kip1. Moreover, in order to investigate whether miR-196a regulated cell growth in laryngeal cancer cells by targeting p27kip1, rescue studies were performed in laryngeal cancer cells.

**Results:**

Results showed that overexpression of p27kip1 rescue decreased cell proliferation caused by miR-196a inhibitors. A negative relation between miR-196a and p27kip1 expression in laryngeal cancer tissues were also noted by further analyses.

**Conclusions:**

The present study showed that miR-196a was upregulated in laryngeal cancer and promoted cell proliferation by downregulating p27kip1 in laryngeal cancer. However, further studies are needed to verify this finding.

## Background

Laryngeal squamous cell carcinoma (LSCC) is one of the most common cancers in incidence and mortality in the head and neck areas [1]. The dismal outcome of patients with laryngeal cancer has been attributed to late diagnosis, recurrence, and metastasis. So far, surgical removal or radiotherapy remains the mainstay treatment of laryngeal cancer, although it is curable when found and treated in the early phase. The prognosis for patients in the advanced stage is still very poor [2]. In China, the 30-year survival rates of patients with laryngeal cancer has improved moderately or even decreased in part due to the relatively high local recurrence [3]. Hence, there is a pressing need to identify both novel highly sensitive biomarkers and new targets for therapeutic intervention for laryngeal cancer which may aid the diagnosis and improve patient outcomes.

MicroRNA (miRNA), encoding a small non-coding RNA of 20–22 nucleotides, is now recognized as a large gene family expressed in plants, animals, and viruses as well as in unicellular algae [4]. It acts as post-transcriptional regulators by inhibiting gene expression through either cleavage of the target mRNA or translational repression [5]. The role of miRNAs in disease processes has received greater attention in recent years due to their capability of regulating a multitude of genes [6]. Naturally, the aberrant expression of miRNA in the pathogenesis of various cancer types has been documented [7–11]. Some studies have demonstrated that miRNAs are often significantly down-regulated in cancers and have the potential to act as tumor suppressors [7, 12], while others have shown that miRNAs can also be up-regulated in tumors and act as oncogenes [13, 14]. For instance, miR-let-7a has been widely found to be down-regulated in several cancers including gastric [15], lung [16], breast [17], and prostate [18]. It functions as a tumor suppressor due to its ability to inhibit oncogene target mRNAs such as Ras [16] and PKM2 [15]. Simultaneously, reports have demonstrated that miR-196a expression is significantly higher in various types of tumors than the controls such as gastric [19], osteosarcoma [20], breast [21], and pancreatic cancers [22, 23]. Since studies have shown that miR-196a contributes to the development and progression of cancers, it may be useful as a candidate biomarker for cancer diagnosis [9].

Recently, it has been reported that miR-196a was dramatically up-regulated in laryngeal cancer. Also, miR-196a inhibitor could suppress the laryngeal cancer growth in vivo or in vitro [9]. However, the regulatory function that miR-196a plays in laryngeal carcinoma, although elucidating the biologic consequences of miRNA dysregulation and identifying the targets of miRNAs, is critical to understanding miRNA pathways and their underlying molecular mechanisms. Recently, it has been reported that miR-196a promotes cancer cells proliferation by downregulating p27^kip1^ in gastric cancer [19]. It is of interest to investigate if similar events have occurred in laryngeal cancer.

In the present study, we conducted a qPCR analysis of miR-196a expression in human laryngeal cancer tissues and showed that miR-196a was overexpressed in tumor-derived samples and laryngeal cancer cell lines compared with matched normal controls. Further functional analysis of miR-196a demonstrated that the inhibition of miR-196a could inhibit laryngeal cell-cycle progression and proliferation in vitro. Luciferase reporter assay and western blot confirmed that miR-196a may function as an oncogene by directly targeting p27^kip1^. The p27^kip1^-mediated repression in cell proliferation was reverted by exogenous miR-196a expression. A negative relation between miR-196a and p27^kip1^ expression in laryngeal cancer tissues was noted by consecutive further analysis. This is the first report that investigates miR-196a function in laryngeal cancer apoptosis by testing p27^kip1^ expression.

## Methods

### Patients and samples collection

The study was approved by Research Ethics Committee of School of Medicine, Shanghai Jiao Tong University (Shanghai, PR China). Informed consents were obtained from all patients. Laryngeal cancer tissues and normal tissues were obtained from patients who had undergone surgery at Shanghai Ren Ji Hospital. All the tissue samples were collected, immediately snap frozen in liquid nitrogen, and stored at −80 °C prior to RNA extraction.

### Cell lines and culture condition

Human laryngeal squamous cell carcinoma HEp-2 cells and human laryngeal squamous cell carcinoma SNU899 and TU212 were purchased from the Cell Bank of the Chinese Academy of Sciences. HEK293T cells for the luciferase reporter assay were purchased from the Institute of Biochemistry and Cell Biology (Shanghai). The human normal bronchial epithelial cell line BEAS-2B was obtained from the American Type Culture Collection (Manassas, VA, USA). All cells were cultured in a DMEM medium (Gibco, USA) containing 10 % fetal bovine serum (FBS) (Invitrogen), 100 U/mL penicillin, and 100 lg/mL streptomycin at 37 °C in incubator with 5 % CO_2_.

### Transfection

miRNA mimic and inhibitor transfection laryngeal cancer cells were transfected with miR-196a mimic, miR-196a inhibitor (Applied Biosystems), and Lipofectamine 2000 (Invitrogen) in accordance with the manufacturers’ instructions. Forty-eight hour after the transfection, cells were harvested for further study including western blotting and qPCR analyses.

### RNA extraction and quantitative real-time PCR

Total RNA was extracted from tissues samples and cancer cell lines with TRIzol reagent (Invitrogen). For qPCR RNA was reverse transcribed to cDNA from 100 ng of total RNA by using a Reverse Transcription Kit (Takara). Real-time PCR (RT-PCR) were performed with SYBR Green (Takara). All protocols were carried out according to the manufacturer’s instructions. The primer sequences in terms of GAPDH, miR-196a, and P27^kip1^ were performed according to Sun et al. [19]. Real-time PCR assay was performed on a Step-One Plus Real-Time PCR System (Applied Biosystems, USA), and each RT reaction was performed in triplicate including no-template controls. The reaction was performed in the following conditions: 95 °C for 5 min, followed by 40 cycles of 95 °C for 15 s, and 60 °C for 50 s. The relative quantification of miR-196a and p27^kip1^ were normalized to the expression of U6 and GAPDH using the 2^−ΔΔCT^ method, respectively.

### Western blotting analysis

Total cell protein was abstracted from cells after transfection. The concentration of the protein was measured using a BCA protein assay kit (Beyotime, Shanghai, China) following the manufacturer’s instruction. Samples were electrophoresed by using 10 % SDS-PAGE. The protein was then transferred onto a PVDF (polyvinylidene fluoride) membrane (Bio-Rad, USA). After blocking in skim milk, the membranes were incubated with specific antibodies. Autoradiograms were quantified by densitometry (Quantity One software; Bio-Rad). β-actin was used as the internal reference; goat anti-p27^kip1^ (1:1000) was purchased from Sigma.

### Cell proliferation assays

Cell proliferation assays were conducted using WST assay (Cell Counting Kit-8, Beyotime, Shanghai, China) in accordance with the manufacturer’s instructions.

### Flow cytometry

The HEp-2 and TU212 cells transiently transfected with miR-negative control (miR-NC), miR-196a mimics, miR-196a inhibitors, pEGFP-NC, and stably transfected with pEGFP-p27^kip1^ were harvested 48 h after transfection using trypsinization. Apoptotic cells were detected using an Annexin V/APC and propidium iodide (PI) apoptosis detection kit I (BD Pharmingen, San Diego, CA, USA) in accordance with the manufacturer’s recommendations. The cells were analyzed with a flow cytometry (FACScan; BD Biosciences) equipped with CellQuest software. Cells were distinguished into viable, dead, early apoptotic and apoptotic, and then the relative ratio of early apoptotic cells were compared with the control transfection cells from each experiment. Cells for cell-cycle analysis were stained with propidium iodide for 1 h and analyzed by FACS can flow cytometer and CellQuest 3.3 software. The percentage of the cells in G0/G1, S and G2/M phase were counted and compared [24].

### Luciferase reporter assay

MiR-196a mimics, miRNA normal control (miR-nc), and miR-196a inhibitors were purchased from GenePharma (Shanghai, China). All transfections were performed using Lipofectamine 2000 (Invitrogen, USA) according to the manufacturers’ instructions. HEK 293T cells were placed into 48-well plates and then cotransfected with miR-196a inhibitor (200 ng) and either luciferase reporter plasmids (200 ng) containing wild-type (WT) or mutant type (Mut) of p27^kip1^ 3′UTR. Forty-eight h after transfection, luciferase activities were measured using the dual-luciferase reporter assay system. The relative reporter activity was normalized by r-luc activity.

### Statistical analysis

The data are presented as mean ± SD. The Students test and one-way ANOVA were used to conduct the comparison of the different protein, mRNA, luciferase reporter, and miRNA expression levels. Statistical analyses were performed using SPSS 16.0 software. A p value less than 0.05 was considered statistically significant.

## Results

### MiR-196a expression is upregulated in human laryngeal cancer tissues and cell lines

**Fig. 1:**
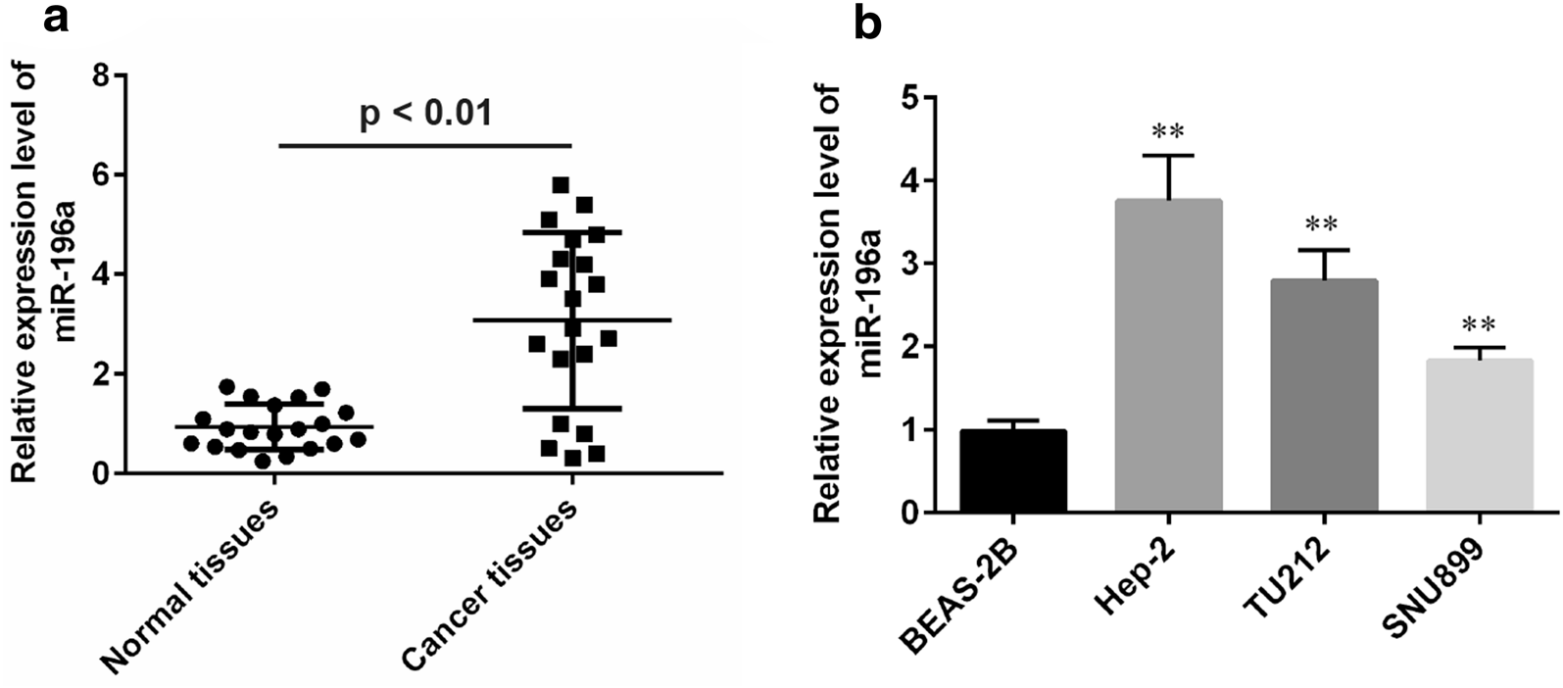
Expression levels of miR-196a in laryngeal tissues and cell lines by real-time PCR analysis. **a** MiR-196a expression was examined by qRT-PCR in 20 laryngeal tissues and in 20 adjacent noncancerous counterparts (NCs) MiR-196a was significantly higher in laryngeal tissues than their counterparts. **b** MiR-196a was also up-regulated in Hep-2, TUU212, and SNU899 laryngeal cell lines compared with normal cell line (**b**). *p < 0.05; **p < 0.01

In the present study, qRT-PCR analyses were conducted to measure the levels of miR-196a expression in 20 laryngeal cancer tissues and three laryngeal cancer cell lines compared with normal counterparts. The expression of miR-196a in cancer tissues was significantly higher than their matched normal tissue samples (p < 0.01), and was also significantly up-regulated in Hep-2 (p < 0.01), TU212 (p < 0.01), SNU899 (p < 0.01) laryngeal cancer cells lines as compared with the normal cell lines (Fig. 1a, b). Among the three laryngeal cell lines, the miR-196a expression was the highest in Hep-2, followed by TU212 cell lines and therefore used for further study.

### Exogenous downregulation of miR-196a in laryngeal cancer cells

**Fig. 2:**
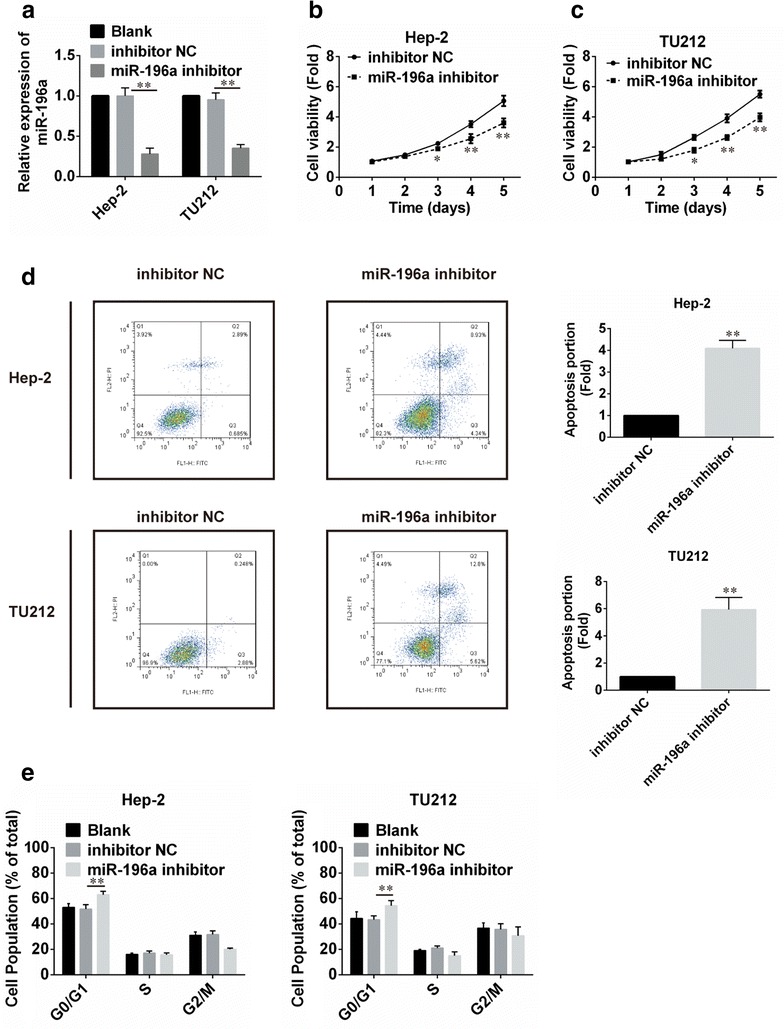
Effect of miR-196a on cell proliferation and apoptosis. **a** The relative expression level of miR-196a in Hep-2 and TU212 cells, transfected with miR-196a negative control (miR-NC) or inhibitors, was tested by qPCR. **b** and **c** WST assay was conducted to detect the proliferation of Hep-2 cells and TU212 cells, respectively. **d** The apoptotic rates of cells were detected by flow cytometry. **e** The *bar chart* represents the percentage of cells in G0/G1, S, or G2/M phase, as indicated. *p < 0.05; **p < 0.01

In order to downregulate the expression of miR-196a, miR-NC, and miR-196a inhibitors were transiently transfected into Hep-2 and TU212 cells, respectively. Our data showed that the miR-196a was significantly down-regulated in both Hep-2 and TU212 cell lines following transfection with miR-196a inhibitors when compared with the negative control and blank (Fig. 2a).

### Effect of miR-196a on cell proliferation and apoptosis in vitro

To evaluate the impact of miR-196a on laryngeal cancer cell proliferation, WST assay was conducted to detect cell viability. We observed significant suppression of cell proliferation in Hep-2 (Fig. 2b) and TU212 cell lines that were treated with the miR-196a inhibitor compared with the control (Fig. 2c), respectively.

In addition, to determine whether cell proliferation was influenced by apoptosis, the quantification of the cell numbers was analyzed by flow cytometric analysis. Results showed that more apoptotic cells were present in both Hep-2 and TU212 cells after treatment with the miR-196a inhibitor as compared with the control (Fig. 2d). To further examine whether the effect of miR-196a on proliferation of laryngeal cells reflected cell-cycle arrest, cell-cycle progression was analyzed by flow cytometric analysis. The results revealed that both Hep-2 and TU212 cell lines transfected with miR-196a inhibitors had an obvious cell-cycle arrest at the G0/G1phase (Fig. 2e).

### miR-196a directly targets p27^kip1^ in laryngeal cancer cell lines

**Fig. 3:**
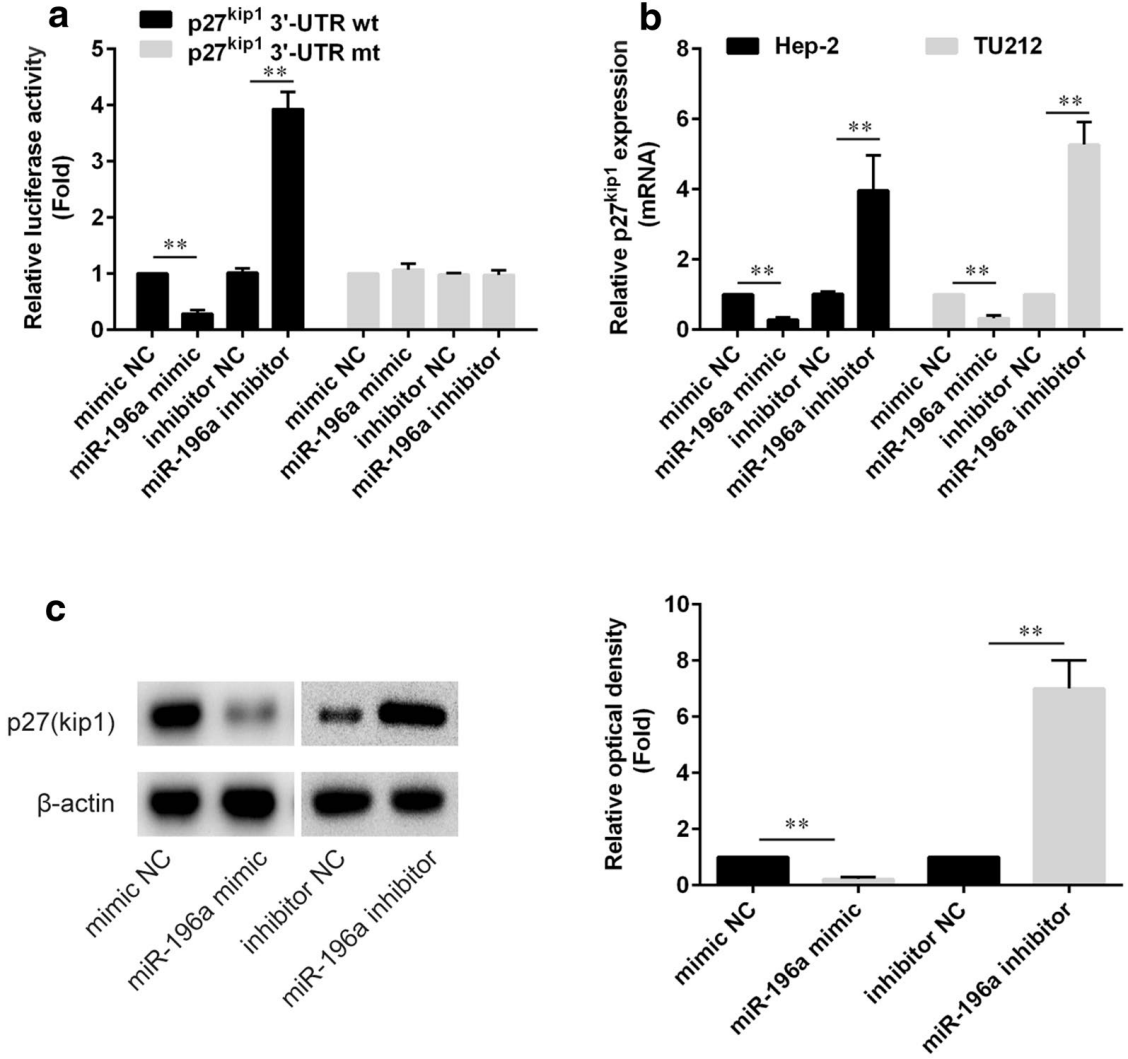
miR-196a directly targets the p27^kip1^ gene. **a** The luciferase reporter plasmid containing wild-type or mutant p27^kip1^ 3′-UTR was cotransfected into HEK-293T cells with miR-196a mimic or mimic NC. Luciferase activity was determined by the dual luciferase assay and shown as the relative firefly activity normalized to Renilla activity. **b** The level of p27^kip1^ mRNA was determined by qPCR. **c** the expression of p27^kip1^ protein was analyzed by Western blotting. β-actin was used as control. *p < 0.05; **p < 0.01

We cloned the wild-type 3′-UTRs of p27^kip1^ gene and inserted it into the region immediately downstream of a luciferase reporter gene. Subsequently, miR-196a inhibitor or miR-196a mimics were transfected with different luciferase 3′-UTR constructs into HEK293T cells. We found that miR-196a significantly decreased the relative activity of the luciferase reporter containing the wild-type 3′-UTR of p27^kip1^ mRNA (p < 0.01; Fig. 3a). To test whether p27^kip1^ mRNA is the target for miR-196a, we mutated the predicted binding site of miR-196a in the 3′-UTR. However, luciferase activity did not sharply drop in the UTRs with mutant-binding sites when compared with Mut-type counterparts (Fig. 3a).

**Fig. 4:**
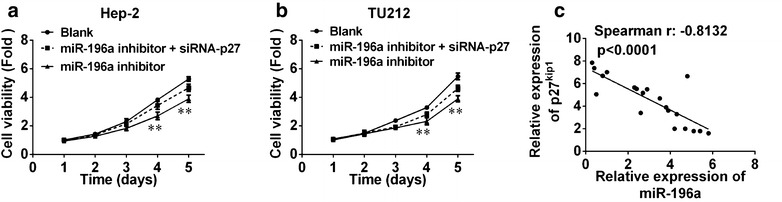
miR-196a regulated cell proliferation through targeting p27^kip1^. **a**, **b** siRNA of p27^kip1^ rescue decreased cell profileration caused by miR-196a inhibitors in Hep-2 (**a**) and TU212 (**b**) cell lines, respectively. **c** An inverse relationship between the expression of p27kip1 and miR-196a was detected

Furthermore, we determined whether miR-196a could regulate p27^kip1^ at both mRNA and protein levels. qPCR analyses showed that the expression of p27^kip1^ mRNA in Hep-2 cells transfected with miR-196a inhibitor or mimics was upregulated or downregulated compared with cells transfected with control, and similar events occurred in TU212 cells (Fig. 3b). Western blot analysis showed that the expression of p27^kip1^ protein in Hep-2 and TU212 cells transfected with miR-196a inhibitor was upregulated compared with cells transfected with negative control (Fig. 4c). These data showed that miR-196a could regulate p27^kip1^ at both mRNA and protein levels.

### Silencing of p27kip1 rescue decreased cell proliferation caused by miR-196a inhibitor

Moreover, to investigate whether p27^kip1^ was involved in the miR-196a–induced increase in laryngeal cancer cell proliferation, we carried out rescue experiments. After transfection with miR-196a inhibitors, Hep-2 and TU212 cells were co-transfected with si-p27^kip1^, respectively. By WST-8 assay, we observed that co-transfection of si-p27^kip1^ and miR-196a inhibitors could partially rescue miR-196a inhibitors–decreased proliferation in Hep-2 (Fig. 4a) and TU212 cells (Fig. 4b), respectively.

### Inverse relationship between the expression of p27^kip1^ and miR-196a

To assess the relationship between p27^kip1^ and miR-196a expression in laryngeal cancer, we examined p27^kip1^ and miR-196a by qPCR in 20 pairs of laryngeal cancer tissues. The results showed that p27^kip1^ mRNA expression was generally lower in laryngeal cancer tissues when compared with matched normal tissues. Further analyses indicated that expression of miR-196a expression was negatively correlated with p27^kip1^ protein level in laryngeal cancer (Fig. 4c). These data suggested that the p27^kip1^ level was mostly opposite to miR-196a expression in laryngeal cancer. These data suggested that miR-196a promotes laryngeal cell proliferation through downregulation of p27^kip1^ expression.

## Discussion

It has been demonstrated that miRNAs may act as activators or inhibitors of tumor proliferation and metastasis [25]. MiR-196a has been identified as an oncogene and reported up-regulated in some tumors include laryngeal cancer [19–21, 23, 26]. However, no information is available on the mechanism that altered miR-196a effect on the growth of laryngeal cancer cell.

We found that miR-196a was dramatically up-regulated in laryngeal cancer tissues and cells, which suggests that high expression of miR-196a might be involved in laryngeal carcinogenesis. The results obtained herein is consistent with the report by Saito et al. who found that miR-196a was highly expressed in the laryngeal cancer cell line JHU-011 and was also more apparent in laryngeal cancer tissues than normal controls [9]. It is a common that increased cell proliferation occurs during laryngeal malignant proliferation. Herein, we raised the possibility that miR-196a might positively regulate laryngeal cell proliferation and thereby may serve as an aid for the diagnosis of laryngeal cancer.

In order to further investigate the function of miR-196a in laryngeal cells, we detected the viability and proliferation of both Hep-2 and TU212 cells. The results of a WST assay showed that viability and proliferation of Hep-2 and TU212 cells were significantly lower in cells transfected with miR-196a inhibitors than those in cells transfected with miR-NC. Saito et al. found that the inhibition of miR-196a hindered cancer cell proliferation in laryngeal cancer-derived cells [9], which is in keeping with our findings. A study reported by Sun et al. demonstrated that overexpression of miR-196a resulted in a marked increase in proliferation of gastric cancer [19]. Based on our results and those of the previously cited studies, it appears that miR-196a could improve the viability and proliferation of laryngeal cancer cells (Hep-2 and TU212).

In addition to inhibiting cell proliferation, we found that miR-196a inhibitor has the effect of promoting apoptosis, in terms of Hep-2 and TU212 cells. A recent report on lung cancer revealed that inhibition of miR-196a could suppress cancer cell lines A549/DDP cell proliferation through the induction of cell apoptosis [27]. Cell proliferation in laryngeal cancer-derived cells transfected inhibition of miR-196a might be result directly from cell apoptosis.

We carried out further studies to detect the cell cycle in Hep-2 and TU212 cells transfected with miR-196a inhibitors. The results of the cell cycle assay by flow cytometry indicated that miR-196a inhibitor could induce cell cycle progression of Hep-2 and TU212 cells arrest at the G1–S phase. To some extent, our observation was consistent with findings in gastric cancer cells, which showed that overexpression of miR-196a could promote gastric cell-cycle progression and proliferation in vitro and in vivo [19].

It has been demonstrated that the cyclin-dependent kinase (CDK) inhibitor p27^kip1^ is a critical regulator of the G1-S transition of the cell cycle and also regulates microtubule (MT) stability [28]. The Cdk inhibitor p27^Kip1^ is present at high levels in quiescent cells and is suppressed in tumor cells [29]. Therefore, it is known as a tumor suppressor gene and a fundamental negative regulator of cell cycle progression [30]. Recently, studies have shown that p27^kip1^ controls the early stage of G1/S phase transition, which may be particularly relevant in the context of tumor progression [31]. Prior to that, it has been reported that miR-196a is up-regulated in gastric cancer and promotes cell proliferation by downregulating p27^kip1^ [19]. miR-196a is also observed with up-regulated expression levels in laryngeal cancer. Therefore, it is important to determine which gene it targets. To better understand the mechanism of miR-196a in laryngeal cancer, we performed luciferase reporter assay, real-time PCR, and western blot to confirm p27^kip1^ is the target of miR-196a in laryngeal cancer cells.

We also observed that p27^kip1^ expression is downregulated in laryngeal cancer tissues and cell lines and negatively correlated with miR-196a expression levels. In addition, further experiments showed that decreased cell proliferation caused by miR-196a inhibitors could be rescued by concomitant overexpression of p27^kip1^, which contributes to the increased understanding of the role of miR-196a in laryngeal cancers. Therefore, it is concluded that miR-196a was upregulated in laryngeal cancer and promoted cell proliferation by downregulating p27^kip1^ in laryngeal cancer.

However, there were also some limitations in our study. Previous studies have demonstrated that one miRNA could alter the expression of a number of genes, while one gene could also be regulated by multiple miRNAs [25]. Thus, there might be other target genes regulated by miR-196a involved in the proliferation of laryngeal cancer cells. Future research should address these issues. Also, further studies regarding the relationship between miR-196a expression and tumor growth are also needed to develop a better understanding of miR-196a in the progression of laryngeal cancer.

## Abbreviations

LSCC: laryngeal squamous cell carcinoma
RT-PCR: real-time PCR
miR-nc: miRNA normal control
miR-NC: miR-negative control

## References

[1] Chu EA, Kim YJ (2008). Laryngeal cancer: diagnosis and preoperative work-up. Otolaryngol Clin North Am.

[2] Sethi N, Rafferty A, Rawnsley T, Jose J (2015). Short, sharp shock public health campaign had limited impact on raising awareness of laryngeal cancer. Eur Arch Otorhinolaryngol.

[3] Li PJ, Hu WH, Jin T (2016). Management of the N0 neck in recurrent laryngeal squamous cell carcinoma. Mol Clin Oncol.

[4] Witold F, Bhattacharyya SN, Nahum S (2008). Mechanisms of post-transcriptional regulation by microRNAs: are the answers in sight?. Nat Rev Genet.

[5] Bartel DP (2004). MicroRNAs: genomics, biogenesis, mechanism, and function. Cell.

[6] Bayoumi AS, Sayed A, Broskova Z, Teoh JP, Wilson J, Su H, Tang YL, Kim IM (2016). Crosstalk between long noncoding RNAs and MicroRNAs in health and disease. Int J Mol Sci.

[7] Calin GA, Croce CM (2006). MicroRNA signatures in human cancers. Nat Rev Cancer.

[8] Sun M, Liu XH, Li JH, Yang JS, Zhang EB, Yin DD, Liu ZL, Zhou J, Ding Y, Li SQ, Wang ZX, Cao XF, De W (2012). MiR-196a is upregulated in gastric cancer and promotes cell proliferation by downregulating p27 (kip1). Mol Cancer Ther.

[9] Saito K, Inagaki K, Kamimoto T (2013). MicroRNA-196a is a putative diagnostic biomarker and therapeutic target for laryngeal cancer. PLoS ONE.

[10] Fu SW, Lee W, Coffey C, Lean A, Wu X, Tan X, Man YG, Brem RF (2016). miRNAs as potential biomarkers in early breast cancer detection following mammography. Cell Biosci..

[11] Kwan JY, Psarianos P, Bruce JP, Yip KW, Liu FF (2016). The complexity of microRNAs in human cancer. J Radiat Res..

[12] Hammond SM (2006). MicroRNAs as oncogenes. Curr Opin Genet Dev.

[13] Zhang B, Pan X, Cobb GP, Anderson TA (2007). microRNAs as oncogenes and tumor suppressors. Dev Biol.

[14] Hammond SM (2006). MicroRNAs as oncogenes. Curr Opin Genet Dev.

[15] Tang R, Yang C, Ma X (2016). MiR-let-7a inhibits cell proliferation, migration, and invasion by down-regulating PKM2 in gastric cancer. Oncotarget..

[16] Wang YY, Ren T, Cai YY, He XY (2013). MicroRNA let-7a inhibits the proliferation and invasion of nonsmall cell lung cancer cell line 95D by regulating K-Ras and HMGA2 gene expression. Cancer Biother Radiopharm..

[17] Yu F, Yao H, Zhu P (2007). let-7 regulates self renewal and tumorigenicity of breast cancer cells. Cell.

[18] Dong Q, Meng P, Wang T, Qin W, Qin W, Wang F, Yuan J, Chen Z, Yang A, Wang H, Aziz SA (2010). MicroRNA let-7a inhibits proliferation of human prostate cancer cells in vitro and in vivo by targeting E2F2 and CCND2. PLoS ONE.

[19] Sun M, Liu XH, Li JH (2012). MiR-196a is upregulated in gastric cancer and promotes cell proliferation by downregulating p27(kip1). Mol Cancer Ther.

[20] Shang Y, Wang LQ, Guo QY, Shi TL (2015). MicroRNA-196a overexpression promotes cell proliferation and inhibits cell apoptosis through PTEN/Akt/FOXO1 pathway. Int J Clin Exp Pathol..

[21] Han Q, Zhou C, Liu F, Xu G, Zheng R, Zhang X (2015). MicroRNA-196a post-transcriptionally upregulates the UBE2C proto-oncogene and promotes cell proliferation in breast cancer. Oncol Rep.

[22] Mark B, Frankel WL, Fabio P (2007). MicroRNA expression patterns to differentiate pancreatic adenocarcinoma from normal pancreas and chronic pancreatitis. JAMA.

[23] Papaconstantinou IG, Lykoudis PM, Gazouli M, Manta A, Polymeneas G, Voros D (2012). A review on the role of microRNA in biology, diagnosis, and treatment of pancreatic adenocarcinoma. Pancreas.

[24] Yang F, Sarangarajan R, Le Poole IC, Medrano EE, Boissy RE (2000). The cytotoxicity and apoptosis induced by 4-tertiary butylphenol in human melanocytes are independent of tyrosinase activity. J Invest Dermatol.

[25] Nicoloso MS, Spizzo R, Shimizu M, Rossi S, Calin GA (2009). microRNAs–the micro steering wheel of tumour metastases. Nat Rev Cancer.

[26] Bloomston M, Frankel WL, Petrocca F (2007). MicroRNA expression patterns to differentiate pancreatic adenocarcinoma from normal pancreas and chronic pancreatitis. JAMA.

[27] Li JH, Luo N, Zhong MZ, Xiao ZQ, Wang JX, Yao XY, Peng Y, Cao J (2015). Inhibition of microRNA-196a might reverse cisplatin resistance of A549/DDP non-small-cell lung cancer cell line. Tumour Biol.

[28] Sherr CJ, Roberts JM (1995). Inhibitors of mammalian G1 cyclin-dependent kinases. Genes Dev.

[29] Hashemolhosseini S, Nagamine Y, Morley SJ, Desrivières S, Mercep L, Ferrari S (1998). Rapamycin inhibition of the G1 to S transition is mediated by effects on cyclin D1 mRNA and protein stability. J Biol Chem.

[30] Fero ML, Rivkin M, Tasch M, Porter P, Carow CE, Firpo E, Polyak K, Tsai LH, Broudy V, Perlmutter RM (1996). A syndrome of multiorgan hyperplasia with features of gigantism, tumorigenesis, and female sterility in p27(Kip1)-deficient mice. Cell.

[31] Berton Stefania, Pellizzari Ilenia, Fabris Linda, D’Andrea Sara, Segatto Ilenia, Canzonieri Vincenzo, Marconi Daniela, Schiappacassi Monica, Benevol Sara, Gattei Valter, Colombatti Alfonso, Belletti Barbara, Baldassarre Gustavo (2014). Genetic characterization of p27kip1 and stathmin in controlling cell proliferation in vivo. Cell Cycle.

